# Pyrazole-derived TRPC3 antagonist ameliorates synaptic dysfunctions and memory deficits in Alzheimer’s disease models

**DOI:** 10.1038/s41380-026-03592-6

**Published:** 2026-04-11

**Authors:** Jiaxing Wang, Ling Chen, Zhengjun Wang, Xing-Yong Chen, Sicheng Zhang, Dongyi Ding, Yuyang Zhou, Ryan Rager-Aguiar, Geng Lin, Hua Zhang, Vijay K. Boda, Tyler C. Ortyl, Peter T. Nelson, Ilya Bezprozvanny, Fu-Ming Zhou, Jianyang Du, Zhongzhi Wu, Wei Li, Francesca-Fang Liao

**Affiliations:** 1 Pharmaceutical Sciences, College of Pharmacy, The University of Tennessee Health Science Center, Memphis, TN 38163 USA; 2 Department of Pharmacology, Addiction Science and Toxicology, The University of Tennessee Health Science Center, Memphis, TN 38163 USA; 3 Department of Cell Biology and Genetics, The School of Basic Medical Sciences, Fujian Medical University, Fuzhou, 350001 P. R. China; 4 Department of Neurology, Shengli Clinical Medical College of Fujian Medical University, Fuzhou University Affiliated Provincial Hospital, Fuzhou, 350001 P. R. China; 5 Department of Physiology, University of Texas Southwestern Medical Center, Dallas, TX 75390 USA; 6 Department of Pathology and Laboratory Medicine, College of Medicine, University of Kentucky, Lexington, KY 40536 USA; 7 Laboratory of Molecular Neurodegeneration, Peter the Great St Petersburg State Polytechnical University, St Petersburg, Russian Federation 195251 Russia; 8 Anatomy and Neurobiology, The University of Tennessee Health Science Center, Memphis, TN 38163 USA

**Keywords:** Neuroscience, Molecular biology

## Abstract

Transient receptor potential canonical (TRPC) channels are widely expressed in the brain; however, their precise roles in neurodegenerative diseases, such as Alzheimer’s disease (AD), remain elusive. We found that TRPC3 expression is upregulated in excitatory neurons of brains with AD. We tested a selective inhibitor (JW-65) for TRPC3 over TRPC6 to investigate the potentially distinct role of TRPC3 in AD. JW-65 treatment significantly restored impaired synaptic plasticity and learning memory in acute and chronic experimental AD models. JW-65 treatment of late symptomatic 5XFAD transgenic mice reversed the impaired LTP, correlating with their significantly corrected synaptic gene expression based on hippocampal RNA-seq data analysis. JW-65 also provided synaptic protection in primary rat hippocampal neurons against soluble β-amyloid oligomers (AβOs), primarily via restoring the AβOs-impaired Ca^2+^/calmodulin-mediated signaling pathways. JW-65 treatment also significantly prevented Ca^2+^ overload induced by AβOs. These findings suggest that aberrantly upregulated TRPC3, as a novel non-selective ion channel, significantly contributes to Ca^2+^ dyshomeostasis in AD. Our work identifies TRPC3 as a potential therapeutic target for treating or preventing synaptic dysfunction of AD.

## Introduction

Alzheimer’s disease (AD) is a progressive neurological condition characterized pathologically by extracellular plaques consisting of β-amyloid (Aβ) and intracellular neurofibrillary tau-tangles, as well as neuronal loss and neuroinflammation. AD manifests significant cognitive decline, which primarily affects memory. Genetic mutations in genes encoding for amyloid precursor protein or presenilin are associated with early onset of AD, resulting in increased Aβ production and disrupted intracellular Ca^2+^ homeostasis. This was the initial fundamental evidence supporting the two major hypotheses of AD pathogenesis: the amyloid cascade hypothesis [1, 2] and the Ca^2+^ hypothesis [3, 4]. However, the channels involved in disrupted Ca^2+^ homeostasis are not fully identified. One of the FDA-approved AD drugs, memantine, is a non-competitive antagonist to NMDA receptor/NR2B, and can only relieve symptoms in mild AD patients, implying that other types of Ca^2+^ channels may also play a significant role in AD pathogenesis.

Ca^2+^ homeostasis is crucial for neuronal functions, which include development, survival, synaptic transmission, memory, and plasticity. Cellular Ca^2+^ signals are regulated by the entry of Ca^2+^ from the extracellular environment or the release of Ca^2+^ from intracellular compartments through receptor/store-operated Ca^2+^ entry (ROCE/SOCE) mechanisms [5, 6]. The transient receptor potential canonical (TRPC) family consists of seven members of the Ca^2+^-permeable nonselective cationic membrane channels within the TRP superfamily [7]. Other than TRPC2, which is encoded by a pseudogene in humans, the two TRPC1/4/5 and TRPC3/6/7 subfamily members are largely expressed in the central nervous system (CNS), especially in the developing cerebellum and hippocampus [8–10]. Given the increasingly recognized roles of the TRPC family members in multiple age-related diseases [11], they have been widely considered as potential drug targets [12], including for neurodegenerative diseases. However, their roles in chronic neurodegeneration are relatively understudied [13] compared to those in acute CNS conditions which have been assessed experimentally in mouse models [14–16].

Among the TRPC family, TRPC6’s neuroprotective roles have been the most studied [17–19] including in several mouse models of familial AD. Overexpression of TRPC6 enhances spatial learning abilities in APP/PS1 mice partially via reducing the production and deposition of Aβ [20], but also by Aβ-impaired Ca^2+^ entry, as well as synaptic dendritic morphology and function in APP-KI models [21]. Moreover, reduced TRPC6 levels were detected in AD patients’ sera [22, 23]. Even though these two closely related subfamily members TRPC3 and TRPC6 were often reported as TRPC3/6 to play similar or overlapping roles from earlier research [17], evidence is emerging more recently suggesting that TRPC3 may play a distinct role from TRPC6 in several experimental settings. For example, they display opposite expressional changes (e.g., upregulated TRPC3 and downregulated TRPC6) in rat hippocampus after seizure [24, 25], consistent with the originally identified pivotal roles of TRPC3 channels in the maintenance of cell excitability and induction of membrane depolarization [5, 26]. Of note, overactivated TRPC3 channels were also reported to cause maldeveloped cerebellar Purkinje neurons, underlying the ataxia phenotype in the moonwalk mice [27, 28]. Furthermore, overactivated TRPC3 channels negatively regulated hippocampal excitability via modulating the Ca^2+^-dependent afterhyperpolarization [29]. Altogether, we speculated that TRPC3 plays a significant role in AD pathogenesis as in many other age-related diseases [30]. Although a pyrazole-based compound (Pyr3) is widely regarded as a TRPC3-selective antagonist [31], its poor stability in blood limits its use in in vivo studies. Therefore, we developed a pharmacological TRPC3 selective antagonist compound with more feasible CNS permeability and stability [32] to investigate the potentially important role(s) of aberrant TRPC3 functions in AD pathogenesis in both in vitro and in vivo experiments.

Over the past two decades, soluble oligomeric forms of Aβ (AβOs) have been recognized as a major culprit targeting synaptic components [33], including Ca^2+^ disruption [34], in AD. NMDA receptors are widely accepted as the principal membrane molecules responsible for Ca^2+^ overload that leads to neurotoxicity and neuronal death [35]. Potential contributions from the TRPC family have never been addressed in response to Aβ regarding Ca^2+^ signaling. Here, we report that AβOs induce *Trpc3* gene upregulation in cultured primary mature neurons that contribute to Ca^2+^ overload and neurotoxicity. Our data demonstrates for the first time that TRPC3 is a key ion channel playing a significant role in mediating Ca^2+^ overload in response to AβOs, including its unique role in intracellular Ca^2+^ handling. Treatment in both acute and chronic experimental models challenged by Aβ, and treated with the selective compound we developed, significantly reverses synaptic dysfunction and memory deficits, identifying TRPC3 as a novel therapeutic target for treating AD.

## Materials and methods

### Study approval

All animal procedures were performed in accordance with the Animal Scientific Procedures Act and with the approval of the Institutional Animal Care and Use Committee (IACUC) at the University of Tennessee Health Science Center. The 5xFAD breeder mice were originally purchased from JAX (Stock #034840) and were bred via mating hemizygous transgenic males with wild-type B6SJLF1 breeders (Jackson Labs, Stock #100012), with the mutant retinal degeneration allele (Pde6b + /rd) bred out of the colony. TRPC3 heterozygous mice (^+/-^) were obtained from (JAX (Stock #016188) and mice were bred between heterozygous to obtain all three genotypes for analysis of P0-1 neurons from the littermates. APPKI mice (*APP*^*NL-G-F*^) were obtained from Dr. Takaomi C. Saito (RIKEN Brain Science Institute, Wakoshi, Saitama, Japan) and maintained via breeding between heterozygous mice.

### Human brain specimens for immunohistochemistry

We used a convenience sample of specimens provided by the University of Kentucky AD Research Center (UK-ADRC) community-based autopsy cohort, and the work performed was approved by the University of Kentucky Institutional Review Board. All samples were obtained from individuals with informed consents at UK-ADRC. Aspects of the UK-ADRC autopsy cohort and biobank, from recruiting to neuropathological workups, have been described previously in detail [36, 37]. From this biobank, 9 human brain prefrontal cortical (PFC; Brodmann Area 9) tissue blocks, collected from short-postmortem interval ( < 6 hr postmortem interval) autopsies, were preserved in formalin, and processed for paraffin-embedding and sectioned at 8 microns for immunostaining. Tissue specimens consist of 4 AD and 5 non-AD controls (NC), many of them with mixed vascular pathology, with the average age of individuals with AD being 85.3 ± 5.6, and NC being 87.0 ± 7.0 (Table S1). All the participants with AD were diagnosed according to a consensus panel of clinicians and pathologists, and were considered sporadic late-onset AD cases. Serial sections were used for staining with antibodies against TRPC3 (Alomone, at 1:1000 dilution), after using Citrate Buffer for antigen retrieval (Sigma, C9999), followed by counter-staining with Hematoxylin, developed using HRP/DAB IHC Detection Kit (Abcam, ab64261).

### Ethic statements

All research procedures used strictly followed relevant guidelines and regulations. For example, human brain samples were collected from individual participant with informed consent and confidentiality, approved by the ethical boards at UK-ADRC to protect the human subjects. All animal work was reviewed and approved by IACUC to protect animal subjects. Other experimental procedure using chemical compounds followed the safe methods approved by the Institutional Safety Board Committee.

### Primary neuronal culture and transfection

Primary cortical and hippocampal neurons were isolated from E17 embryos of Sprague Dawley rats and cultured as described [38–40]. A pregnant female rat was euthanized on E17. The isolated embryos were immediately placed in ice-cold dissection HBSS solution (Thermo Fisher Scientific, 14025076) containing 4 µL/mL glucose (Thermo Fisher Scientific, A2494001), 5 mM HEPES (Thermo Fisher Scientific, 15630130) and 1xPen/Strep (Sigma-Aldrich, P4333). The hippocampus and cortice were further dissected and digested with TrypLE (Thermo Fisher Scientific, 12604013), and DNAase in HBSS dissection buffer with 5 ml 0.25% Trypsin–EDTA supplemented with 150 μL DNase. The dissected tissue samples were incubated for 15 min at 37 °C, washed with HBSS dissection buffer, and spun down in 200 g for 2 min at room temperature (RT). The resulting cells were then seeded at the described densities [40]: Hippocampal neurons were seeded in 24-well-plates (2 × 10^4^ cells per well) on Poly-D-lysine-coated (A3890401, Sigma) coverslips for immunocytochemistry and calcium imaging, and cortical neurons were seeded at 1 × 10^6^ cells per well in 6-well plate or 5 × 10^6^ cells per 100 mm dish for Western blot analyses, respectively. The digested cells were first seeded with a DMEM plating medium (Thermo Fisher Scientific, 11965092) containing (per 500 mL DMEM) 62.5 mL fetal bovine serum (Cytiva HyClone, SH30071.03IR25), 5 mL GlutaMAX (Thermo Fisher Scientific, 35050061), 15.6 mL HEPES (Thermo Fisher Scientific, 15630130), 62.5 mL Ham’s F-12 Nutrient Mix (Thermo Fisher Scientific, 11765054), and 1x Pen/Strep. Two hours later, the plating medium was replaced with Neurobasal culture medium (Thermo Fisher Scientific, 21103049) containing (per 500 mL Neurobasal) 10 mL B-27 supplement (Thermo Fisher Scientific, A1486701), 5 mL GlutaMAX, and 1x Pen/Strep. Fresh culture medium (1:2 replacement of starting volume) was added every 3 days. Transient transfection of primary neurons with siRNAs was performed as described [38] using Lipofectamine for the data presented in Fig. 6: primary neurons were seeded in both 24-well and 6-well plates, and transfected with siRNA3/6/scramble (20 µM stock/40 nM final concentration) mixing with Lipofectamine 3000 (Invitrogen) at ratios of 1:0.6 and 4:2.5 µl, respectively at 5 DIV. After 6 hours of incubation, the cells were replaced with fresh culture medium and harvested 7–9 days after transfection for real-time RT-qPCR.

### siRNA sequences

**Table:** 

	Sense	antisense
siTRPC3	UCAUCUUCCUGGGUCUGCUUGUGUU	AACACAAGCAGACCCAGGAAGAUGA
siTRPC6	GGAGCUCAGAAGAUUUCCAUUUAAA	UUUAAAUGGAAAUCUUCUGAGCUCC

### Naturally secreted AβOs-containing conditioned medium (7PA2 CM), and immunodepletion

The 7PA2 cell line represents Chinese Hamster Ovary (CHO) cells stably transfected with human APP751 which contains a Val717Phe mutation [41]. Conditioned medium (CM) containing naturally secreted AβOs was collected from 7PA2 cells as previously described: 7PA2 and CHO cells were cultured in DMEM media until reaching 80% confluency. Cells were then switched to the Neurobasal medium and cultured for 24 h before collecting the culture supernatant. The collected CM was filtered (Millex, SLG004SL, 0.22 μm) and aliquoted, stored at −80 ^0^C until use. 7PA2 treatment in cultured neurons. We used 1:10 diluted CM in cultured models for overnight (16 h) for the determination of MAP2-stained dendritic analysis, as described [38]. For the experiment described in Fig. S3E, the 7PA2 CM was immunodepleted with anti-Aβ antibody (BAM-10, epitope AA1-10, Sigma) three rounds as described [40] to validate the Aβ-dependent synaptotoxicity in an acute fear contextual memory test.

### Chemical compounds and reagents

JW-65 was synthesized in-house as described [32]. KN92 (4130) and KN93 (1278) were purchased from Tocris Bioscience; RpcAMP from Sigma-Aldrich (A165); LY294002 from LC laboratories (L-7962); U0126 from Cell Signaling Technology (9903S).

### Immunoblotting, immunocytochemistry and immunohistochemistry, RNA isolation, reverse transcription, and RT-qPCR

These procedures were performed as described [38–40, 42]. Images were acquired by Keyence microscope BZ-X800 (Keyence) or Olympus FV1000 confocal laser scanning biological microscope (Olympus Life Science). Quantification of amyloid load, and neuroinflammation were described [42]. Soluble Aβ_42_ levels from the hippocampal tissue of 5xFAD mice (12 months of age) were determined by ELISA kit (294-64701, Wako, Japan), according to the manufacturer’s instructions. Briefly, the mouse hippocampal tissues were homogenized on ice in RIPA buffer (1:10 w/v, Thermo Fisher Scientific, 89900,) containing a protease inhibitor cocktail (Roche, #11836170001), followed by centrifugation at 100,000 × g for 20 min at 4 °C. The supernatant was then collected, and the protein concentration of each sample was determined using a Bio-Rad protein assay kit. The final supernatant was diluted 1000-fold for ELISA analysis. For MAP-2 staining and the subsequent quantification of dendritic length were performed as described [39, 40]. Primers and antibodies used in this research are listed in Table S1-2.

#### Immunoblotting

Cells or brain tissues were homogenized in RIPA buffer (Thermo Fisher Scientific, 89901) and protease inhibitor (Roche, #46931160010) and rested for 30 min on ice. Homogenates were centrifuged at 12,000 g for 20 min at 4 °C. Supernatants were used as total protein lysates and were analyzed by SDS-PAGE (Invitrogen, XP04200BOX). EZ-Run Pre-stained Protein Marker was used for Western blotting (Thermo Fisher Scientific, BP-3601). The antibodies used for Western blotting were TRPC3 (Alomone Labs, ACC-016), TRPC6 (Sigma, SAB4300572), PSD95 (Invitrogen, 51-6900), BDNF (Alomone, ANT-010), cleaved caspase-3 (Cell signaling Technology, 9661), β-actin (Sigma-Aldrich, A2228) and horseradish peroxidase (HRP) conjugated secondary antibodies (Thermo Fisher Scientific, Goat anti-Rabbit, 31460; Goat anti-Mouse, 31430). Blots were transferred onto Immobilon PVDF membranes (Millipore, IPVH00010) were visualized via Gel Doc 2000 Imaging System (Bio-Rad).

#### Immunocytochemistry

Cells were washed with PBS (prepared in house) and fixed with 4% PFA for 20 min. The cells were permeabilized with 0.4% Triton X-100 (Fisher Scientific, BP151-100) in PBS for 5 min, and then blocked with 5% Bovine Serum Albumin (BSA, Miltenyi Biotec, 130-091-376) and 0.4% Triton X-100 (Fisher Scientific, BP151-100) in PBS for 1 hr before incubation with the primary antibody (anti-MAP-2, Sigma 4403, 1:1000), at 4 °C overnight. After washing with PBS, the cells were incubated with DAPI (Invitrogen, D1306) and secondary antibodies for 2 h at room temperature. Finally, the coverslips were mounted using Fluoromount Aqueous Mounting medium (Sigma-Aldrich, F4680).

#### Immunohistochemistry

Deeply anesthetized mice were transcardially perfused with ice-cold saline 4% paraformaldehyde (PFA). Brains were then removed and fixed in 4% PFA for 24 hr at 4 °C and sectioned using Leica VT 1200S vibratome (Leica Biosystems, 30–50 μm). The brain slices were blocked, stained with primary and secondary antibodies, and finally mounted as described for immunocytochemistry. Images were acquired by Keyence microscope BZ-X800 (Keyence) or Olympus FV1000 confocal laser scanning biological microscope (Olympus Life Science).

#### RT-qPCR

Total RNA was extracted using TRIzol (Thermo Fisher Scientific, 15596026) and quantified by NanoDrop 2000c Spectrophotometer (Thermo Fisher Scientific, ND2000CLAPTOP). Each RNA (500 ng) sample was reverse transcribed into cDNA with High-Capacity cDNA Reverse Transcription Kit (Thermo Fisher Scientific, 4368814). RT-qPCR was performed on Mastercycler Realplex Real-time PCR System (Eppendorf) using SsoAdvanced Universal SYBR Green Supermix (Bio-Rad, 1725270). The reaction used white 96-well plates (Azenta, 4ti-0741). The quantity of mRNA was detected with SYBR Green supermix (1725271, Bio_Rad) and LightCycler Instrument (Roche) and quantified by 2-ΔΔCt calculation based on the triplicate Ct values normalized with housekeeping gene Gapdh or β-Actin. Primers were purchased from Invitrogen and used at a concentration of 100 nM.

### In situ hybridization

In situ hybridization was performed as previously described [43]. Briefly, 16-µm tissue frozen sections were fixed in 2% paraformaldehyde in PBS for 10 min, followed by incubation in acetylation with 0.8% triethanolamine and 0.15% acetic anhydride for 10 min, and subsequently permeabilized with proteinase K in PBS for 10 min to allow probe penetration. Pre-treated sections were then hybridized with digoxigenin (DIG)-labeled cRNA probes targeting *Trpc3*, which were generated from a 597-bp cDNA fragment of *Trpc3*. The cDNA fragments (primer_F: CTCGCCATCGGCTATTGGAT, primer_R: CTCGTTTGCAGGGAGGATGT) were cloned and transcribed in vitro using the DIG RNA Labeling Kit (SP6/T7, Roche) to obtain DIG-labeled antisense and sense probes. The antisense probe was used for mRNA detection, while the sense probe served as a negative control. Following stringent post-hybridization washes to remove unbound probe, signals were detected using either goat anti-digoxigenin/digoxin antibody conjugated with DyLight™ 594 (Vector Laboratories, DI-7594-0.5) for fluorescent visualization or anti-digoxigenin-AP (Roche, 11093274910) with NBT/BCIP substrate solution (Roche, 11681451001) for chromogenic detection.

### Dendritic spine analysis in primary hippocampal neuronal cultures (Fig. S2E)

The hippocampal cultures of APPKI and WT mice were established from postnatal day 0-1 pups and maintained as described [21]. For the assessment of synapse morphology, hippocampal cultures were transfected with TD-Tomato plasmid at DIV7 using the calcium phosphate method, and the culture was treated with different concentrations of JW-65 on DIV16. The cultures were then fixed with 4% formaldehyde 24 h later. A-Z-stack of optical section was captured using 100x objective with a confocal microscope (Carl Zeiss Axiovert 100 M with LSM510). Quantitative analysis for dendritic spines from 18–20 neurons each genotype was performed with the NeuronStudio software package [44]. To classify the shape of neuronal spines in culture, we adapted an algorithm from a previously published method [44] for quantification.

### Calcium imaging

The experimental setup (Oregon Green 488 BAPTA-2/AM, Invitrogen, O6809) and the recording conditions were previously described [45]. Extracellular solution was prepared to contain (in mM) 140 NaCl, 6 KCl, 2 CaCl_2_, 1 MgCl_2_, 10 glucose, and 10 HEPES. Divalent-free solution was prepared to contain (in mM) 145 NaCl, 5 EGTA, 2 EDTA Na+ salt, 10 glucose, and 20 HEPES. Oregon Green 488 BAPTA-2/AM (Invitrogen, O6809) was diluted into the solutions at a working concentration of 4–5 µM. Cells were incubated with the above solutions for 1 h. Fluorescent imaging was performed using a Lambda 10-2 Optical Filter Changer Control System (Sutter Instrument) at a sampling rate of 500 ms, recorded by MetaFluor software (Molecular Devices).

### Acute AβOs-induced synaptic toxicity assay- stereotaxic microinjection

Wild-type C57BL/6 mice (2-4 months of age, both sexes) received 2 μl of control (CHO medium), or 7PA2 CM, via an implanted cannula. Surgical implantation and intracerebellar ventricular injections were performed as described [39, 42]. Mice were anesthetized by intraperitoneal injection of ketamine/xylazine (87/13 mg/kg). Analgesic was administered (carprofen, 5 mg/kg). The eyes were applied with eye lubricant and shaded with soft tissue to avoid dehydration over time and damage caused by exposure to the light. The mice were positioned in a stereotaxic mouse frame (Kopf instruments). After being precisely orientated, the skull was exposed, and a hole was drilled into the skull (coordinates to bregma: anteroposterior (AP), −0.37 mm for mice weighing 21–25 g or −0.46 mm for mice weighing 26–30 g; mediolateral (ML), 0.95 mm for mice weighing 21–25 g or 1.00 mm for mice weighing 26–30 g). A guide cannula (Plastics One) designed to reach −1.75 mm dorsoventrally (DV) was mounted to the skull. The scalp, together with the guide cannula, was fixed with superglue or dental cement. A paired dummy cannula (Plastics One) was fitted into the guide cannula to prevent clogging. 1 mL 0.9% NaCl saline was i.p. injected for rehydration. The mice were placed on a heat pad and monitored until complete recovery from anesthesia before returning to the housing cages. 5 mg/kg carprofen was administered QD for the first three days post-surgery. Upon recovery, the dummy cannula was removed and 2 µL CHO/7PA2 CM was injected via an internal cannula (Plastics One) at an injected speed of 0.4 µL/min, controlled by fixing a microinjection syringe (Hamilton) on a syringe pump (Harvard Apparatus). After injection, the internal cannula was kept in place for an additional 5 min and slowly withdrawn to avoid liquid overflow. Finally, the Dummy cannula was placed back. Similar surgical procedure was used to inject AAV2/9-shRNA-mTrpc3 viruses (custom-produced by BioHippo Inc., Gaithersburg, Maryland) intracerebral ventricularly (i.c.v., in 2 μL) to mouse CNS.

### Mouse behavioral tests

Fear conditioning was conducted as described [39, 42]. Fear conditioning consists of two sessions: A training session and a retrieval session. During the training session, each mouse was placed in a chamber (Actimetrics) with a camera on the ceiling and conductive grills as the floor. They were allowed to explore the chamber for 200 s (pre-context). After that, a white noise (80 dB and 2 Hz) followed by a foot shock (0.5 mA) was given as a stimulus two times at 200 s and 280 s, respectively, to the mice (context). Once finished (6 min in total), the mice were placed back in their housing cages. After 24–72 h, the fear retrieval test was performed several times by placing the mice back to the same chamber for 6 min but without stimulus. Freezing behavior during all the sessions was recorded and analyzed by FreezeFrame 3 software (Actimetrics).

The Morris water maze testing paradigm consists of three sessions as we previously described [42]. Spatial learning and memory were tested by the Morris water maze test. A circular tank (1 m in diameter and 40 cm in height) was filled with opaque white water at a depth of 20 cm. Different shape stickers were placed along the walls surrounding the tank as reference cues. An escape platform was placed at the center of one quadrant. A rod with a conspicuous plastic ball on top was fixed vertically on the platform during the cued training session, during which the platform stayed above the water surface. Mouse behavior was video recorded. During cued training, mice were introduced into the pool from four different quadrants, and were allowed to search for the platform for 60 s. If their escape failed, they were guided to the platform and kept on it for 10 s. During hidden platform training, the rod on the platform was removed and the platform was pushed down to be hidden 1 cm underneath the water surface. The same 60 s tests were performed as the cued training test. After training, a probe test was performed by removing the platform and allowing each mouse to swim freely for 60 s. The swimming traces during the three sessions were analyzed via ANY-maze (Stoelting).

### RNA-sequencing analysis

Raw RNA-sequencing analysis was processed with Salmon [46]. Reads were aligned to the Ensembl mouse reference genome GRCm39 and the gene expression was quantified. The raw gene counts were processed in RStudio (Ver2022.07.2 + 576) with DESeq2 package to analyze differentially expressed genes (*p.adj* < 0.05 and Log2 Fold Change (LFC > 0.58).

### Recording of long-term potentiation (LTP) of CA1 field excitatory postsynaptic potentials (fEPSPs) in hippocampal brain slice preparations

The experimental setup (coronal brain slices at 400 µm thickness containing the hippocampus) and the recording conditions were previously described [47]. Mice were deeply anesthetized with isoflurane, and brains were rapidly removed and transferred to ice-cold prep solution containing (in mM) 5 KCl, 1.25 NaH_2_PO_4_, 5 MgSO_4_, 1 CaCl_2_, 205 sucrose, 25 glucose, and 26 NaHCO_3_ (pH 7.4, Osm 300-310) under 95% O_2_ and 5% CO2. Brains were sectioned using Leica VT 1200S vibratome (Leica Biosystems) and incubated in artificial cerebrospinal fluid (ACSF) containing (in mM) 115 NaCl, 2.5 KCl, 2 CaCl_2_, 1 MgCl_2_, 1.25 NaH_2_PO_4_, 25 NaHCO_3_, 11 glucose, and 0.1 vitamin C (pH 7.4, Osm 280-310) under 95% O_2_ and 5% CO_2_ for 2 h at room temperature. Upon recording, a 400-µm slice was transferred into the submerged recording chamber and superfused with ACSF. The Schaffer collateral/commissural fibers in the stratum radiatum were stimulated with a bipolar tungsten electrical stimulating electrode (76 mm in length, 0.5 MΩ nominal impedance. Tungsten, TST33C05KT). Field excitatory postsynaptic potentials (fEPSPs) were recorded from the CA1 stratum radiatum using an extracellular glass pipette filled with ACSF placed 200-300 mm away from the stimulating intensity that yielded 50% of the maximum response. After a 30 min baseline recording, theta-burst stimulation was used to induce LTP, followed by continued recording for 60 min.

### Golgi staining

Golgi staining was performed as previously described [48] using the FD Rapid GolgiStain Kit (PK401, FD Neurotechnologies, Inc., Ellicott City, MD 21041, USA). Spines were quantified with Fiji software [49].

### Statistical analysis

All data are presented as mean ± SD. Sample sizes are described in the figure legends. Statistical analysis was performed using GraphPad Prism (Ver 8.3.0). Two-tailed t-tests were performed for comparisons between two groups, and one- or two-way ANOVAs were performed for more than two groups and multiple comparisons. All tests were conducted as two-sided tests with significance levels set as: ns, *p* > 0.05; *, *p* < 0.05; **, *p* < 0.01, ***, *p* < 0.001; ****, *p* < 0.0001.

## Results

### TRPC3 expression is aberrantly upregulated in autopsy AD brains

**Fig. 1:**
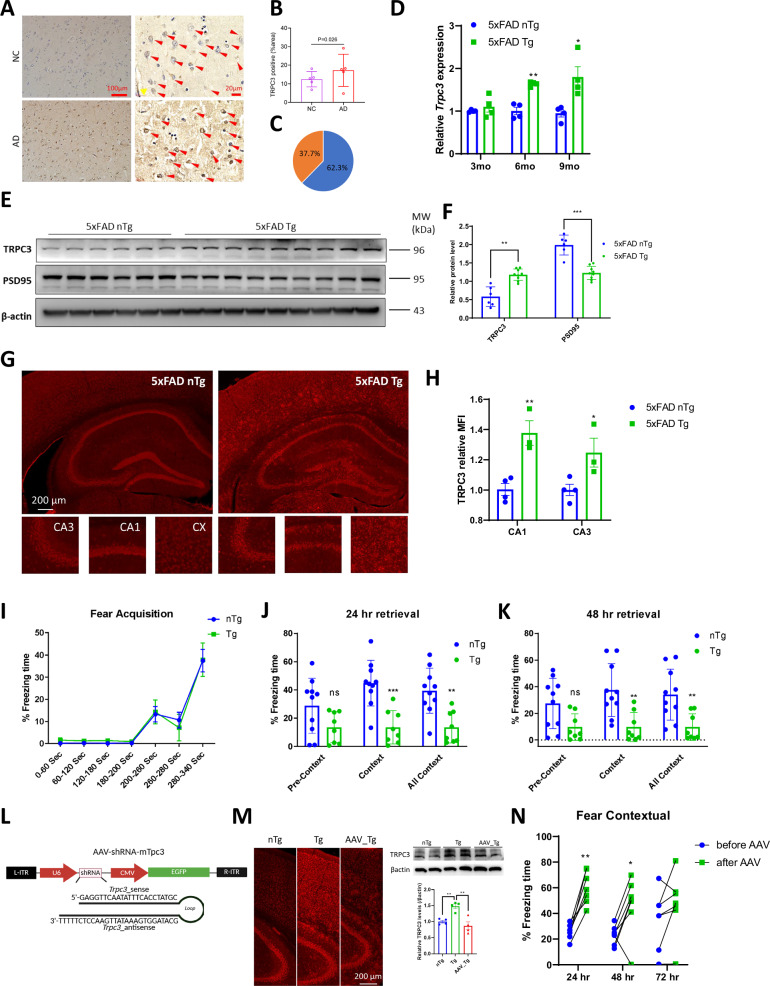
Increased TRPC3 expression in human and mouse AD mice brains. **A-C** Immunohistochemistry (IHC) of the TRPC3 expression in postmortem human AD and non-AD (NC) control patient cortical tissue samples**. A** Representative IHC images showing TRPC3 expression at 200X (left) and 600X (right, 60X oil len) magnifications. Scale bars: 100 μm (200×) and 20 μm (600×), respectively. Red arrowheads indicate positive staining in large pyramidal neurons. **B** Quantitative analysis of TRPC3 expression between AD and NC samples based on 3 images taken from each human tissue case (200x) as described [40]. Data are presented as mean ± SD. **C** The percentages of TRPC3-positive pyramidal neurons (blue) and other cell types (orange) in a pie chart, showing a majority of cells displaying TRPC3 immunosignals are pyramidal neurons (> 60%). Cell counts were performed on images taken under 200X magnification. **D** Increased *Trpc3* mRNA levels in 5xFADTg brain hippocampal tissue detected by quantitative qPCR. N = 3 mice, *, p < 0.05. **, p < 0.01. **E,F** Representative Western blot showing hippocampal protein expressional levels of TRPC3, and PSD95 in 5xFAD mice (10-month-old), with n = 6 for nTg and n = 9 for Tg. Quantification was performed using two-way ANOVA. **, p < 0.01. ***, p < 0.001). **G** Representative images of TRPC3 immunosignals from immunohistochemistry using Abcam’s rabbit antibody (ab51560, 1:1000) of littermate 5xFAD nTg and 5xFADTg mice (8 month-old). **H** Quantified TRPC3 immunosignals from the CA3- CA1 regions based on 3 mouse coronal brain sections of 5xFAD Tg and nTg littermates (8 month-old)). CX: cortex. **I-K** Fear contextual training and 24-48 h fear retrieval test on 9-month-old 5xFAD mice (nTg, n = 10. Tg, n = 8, two-way ANOVA. ns, non-significant. **, p < 0.01. ***, p < 0.001). **L** Schematic design of AAV-shRNA-mTrpc3 viruses**. M** Effect of AAV-shRNA on downregulating TRPC3 expression levels as assessed by Western blots and IHC. Left and right panels show representative images of TRPC3 staining and Western blots, respectively. Quantification graph was based on the Western blots from 2 independent runs. **N** Improved fear contextual memory after AAV viral injection (AAV2/9, Titer: 1.82x10e13 GC/ml, 2 μl injected i.c.v.). Fear contextual memory was performed 4 weeks after viral injection. Statistical analysis: paired t-test;*, p < 0.05, **, p < 0.01.

By immunohistochemistry analysis based on 4 AD cases and 5 non-AD (NC) cases, we detected markedly upregulated TRPC3 expression in AD brains, most notably in the large principle pyramidal neurons (Fig. 1A, red arrowheads), as quantified by total coverage areas (Fig. 1B) as well as the percentages of the immuno-positive cells (Fig. 1C). Considering age as a significant risk factor for both dementia and AD, we examined TRPC3 expression in wild-type (WT) C57BL/6 (B6) mouse brains and found that TRPC3 expression was elevated in an age-dependent manner (Fig. S1A). By immunohistochemistry, we observed widely expressed TRPC3 in the cell bodies located in cortical and hippocampal regions. Enhanced TRPC3 immunosignals were detected in aging mouse brains, most notably in the CA1 and CA3 pyramidal layers, as well as the granule layer of dentate gyrus (DG) (Fig. S1B), which was corroborated by the in situ hybridization patterns (Fig. S1C). We then determined the TRPC3 expressional profile in the most widely used familial AD mouse model of 5xFADTg [50, 51] by a complememtary measurements of quantitative RT-qPCR, Western blots and immunohistochemistry, and observed similarly elevated TRPC3 expression at young age (Fig. 1D–H). On the contrary, the expressions of brain-derived neurotrophic factor (BDNF) and post-synaptic density 95 (PSD95) were reduced in the Tg samples (Fig. 1E, F). Immunohistochemistry revealed similar results based on two commercial antibodies showing markedly increased TRPC3-signals in 5xFAD Tg mice brains at 8 months of age; only the results from one antibody (AB51560, Abcam) are presented (Fig. 1G) which display clustered immunosignals colocalizing with Aβ-immunosignals (Fig. S1D) and mirrored by the Trpc3 mRNA expressional pattern by the in situ hybridization/FISH (Fig. S1E). Interestingly, FISH reveals marked increase in Trpc3 mRNA expression in the white matter (corpus callosum) region in both 5XFAD Tg and PS19 Tg mouse brains, and to lesser degrees in the DG-CA3-CA1 subregions

Based on both the immunohistochemistry and FISH analysis from the same 5xFAD Tg brains, the similar notable clustering pattern of TRPC3 expression not only reflects a high Aβ load in this model but also suggests potential physical interaction between TRPC3 and highly aggregated Aβ. Nevertheless, the increased expression of TRPC3 gene and protein at a young age of 5xFAD Tg mice seems to be in line with the several AD pathological features from this familial model of AD, including synaptic loss, impaired hippocampal long-term potentiation (LTP), and spatial working memory deficits developed around 5-8 months of age [52].

Since hippocampus is most important for synaptic plasticity, and disruption of the excitatory neuronal activity has been shown to contribute to cognitive deficits in AD [53], the increase of TRPC3 expression particularly in excitatory neurons could contribute to AD pathogenesis. We then used a well-established paradigm of fear contextual memory for studying hippocampus-mediated context-dependent behavior facilitated by learning and memory processes. As presented in Fig. 1I–K, comparative analysis with nTg littermates revealed that, during the retrieval test, Tg mice displayed impaired fear contextual memory at 9-months age. Furthermore, administration of the Tg mice intracerebral ventricularly (i.c.v.) with AAV virus-delivered shRNA to mouse Trpc3 gene resulted in significantly enhanced fear contextual memory (Fig. 1L–N). This finding validates TRPC3 as an AD target and provides proof-of-concept of TRPC3 inhibition as a viable therapeutic strategy.

### JW-65 protects against AβOs-induced damage of synaptic structure and signature proteins in vitro

The data presented above supports that TRPC3 upregulation could contribute to AD. Using a primary culture model derived from dissected E17 embryos consisting primarily of hippocampal principal pyramidal excitatory neurons, we tested compounds that inhibit TRPC3 function. Naturally secreted oligomeric AβOs from the 7PA2 cell culture conditioned media (CM) have been well-characterized to exert synaptic toxicity in multiple in vitro and in vivo assays [38–42, 54–56], and the active components were confirmed to be primarily a mixture of oligomeric forms of Aβ (i.e., monomers, dimers, trimers, and other oligomers) [38] in subnanomolar concentrations [57, 58]. Importantly, treatment of cultured mature neurons (13-14 DIV) with 7PA2 CM, but not extracts from CHO cells, significantly impeded neuronal growth in a dose-dependent manner, as indicated by immunostaining of microtubule-associated protein 2 (MAP2), a neuron-specific cytoskeletal protein (Fig. S2A). A ten-fold diluted (10%) 7PA2 CM resulted in consistent neuronal morphological changes and cell death as we previously reported [38–40], and thus was used throughout the subsequent experiments.

**Fig. 2:**
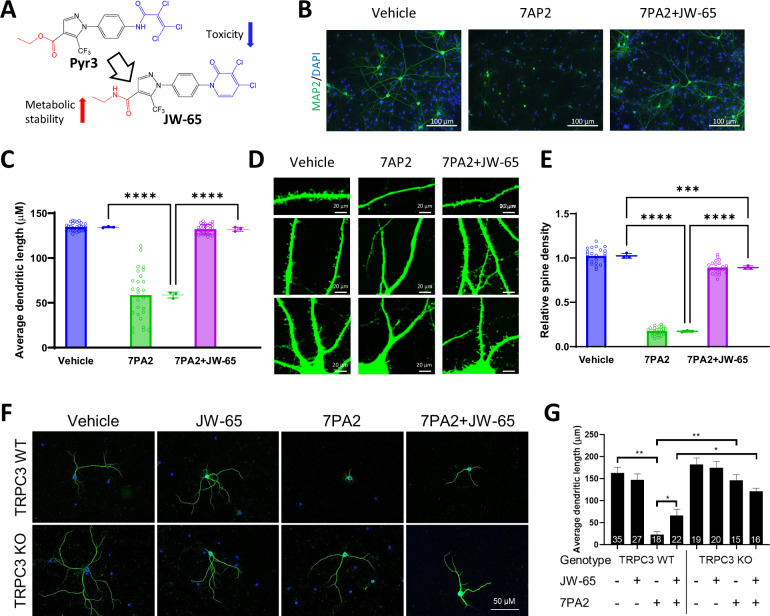
JW-65 protects neurons against 7PA2 treatment. **A** Chemical structure of JW-65 and its prototypic compound Pyr3 [31, 32]. **B**, Representative microscopic images of cultured primary neurons treated with 7PA2 and JW-65 and MAP-2 stained. **C** Quantification of dendritic length. Bar represents all values recorded from three independent experiments/neuronal cultures (n = 30 dendrites for Vehicle, 27 dendrites for 7PA2, and 28 dendrites for 7PA2 + JW-65, respectively), and statistical analysis was based on the mean values from the 3 independent experiments (one-way ANOVA. ****, p < 0.0001). **D** Representative confocal images of dendritic and spine morphology under the treatment of 7PA2 and JW-65 by MAP-2 staining. Scale bars: 20 um. **E** Quantification of spine density. ImageJ 1.48 K version was used to determine dendritic length and spine density was calculated by the total number of spines from each dendrite (n = 24 dendrites for Vehicle, 27 for 7PA2, and 22 for 7PA2 + JW-65, respectively. Graph is presented by using the vehicle group as reference (value 1.0). Data were collected from 3 independent cultures, one-way ANOVA. ***, p < 0.001. ****, p < 0.0001. **F,G** Neurons derived from TRPC3 KO mice are resistant to 7PA2 treatment. **F** Representative MAP-2 microscopic images of mature neurons (21 DIV) derived from the P0-1 pups. **G**, Quantification of dendritic length was based on n ≥ 15 neurons each group, with the number of the included neurons indicated at the bottom of each column.

Our group previously developed JW-65, a brain-penetrative and selective TRPC3 inhibitor that can directly bind to the TRPC3 protein (Fig. 2A) [32]. This compound was optimized based on the scaffold of pyrazole Pyr3, a TRPC3-selective antagonist [31] which inhibits TRPC3 channels with submicromolar (IC_50_ = 0.7 μM) potency and does not inhibit TRPC6 or other TRPCs. However, the poor stability of Pyr3 limits its use for chronic in vivo studies. JW-65 displays improved metabolic stability and systemic toxicity profile over Pyr3 [32]. In cultured neurons, we found that 7PA2 induced upregulated TRPC3 expression [42]. JW-65 co-treatment with 7PA2 restored the TRPC3 protein expressional levels to baseline while JW-65 treatment alone had no effect on the baseline TRPC3 levels (Fig. S2B-C). The neuronal loss induced by 7PA2 was also largely rescued by co-treatment at the optimal concentration of 500 nM as determined in our prior work [32] (Fig. 2B, C). This concentration was used for JW-65 treatment throughout the subsequent in vitro experiments. Furthermore, JW-65 attenuated the 7PA2-induced loss of dendritic spine structure, especially those mushroom spines generally believed to be best correlated with strong synapses and memory storage (Fig. 2D, E) [59–62]. Independent experiments performed on the mature primary neurons isolated from APP-KI mice [21] also demonstrated a significant rescuing effect of JW-65 on the dendritic mushroom spines (Fig. S2D-E).

To determine if JW-65’s synaptic protective effect was mediated through TRPC3, we repeated the 7PA2 experiment in mature neurons derived from the P0-1 pups from TRPC3 KO mouse line. JW-65 treatment in neurons from WT and KO pups, starting from 5 DIV, did not exert significant impact in dendritic morphology. However as shown in Fig. 2F, G, TRPC3 KO neurons were largely resistant to the 7PA2-induced synaptic toxicity based on the quantified dendritic length. Since the TRPC3 deficiency in the KO neurons conferred significant synaptic protection, JW-65 treatment did not result in additional protection. These results suggest the importance of TRPC3 in mediating AβOs-induced synaptic toxicity. Owing to the facts that P0-1 mouse neurons are more difficult to culture and maintain with slower differentiated dendritic morphology, the protective effect of JW-65 on WT mouse neurons was less effective (yet significant) than that on the E17-derived rat neurons.

### JW-65 alleviates memory decline in both acute and chronic β-amyloidosis mouse models

**Fig. 3:**
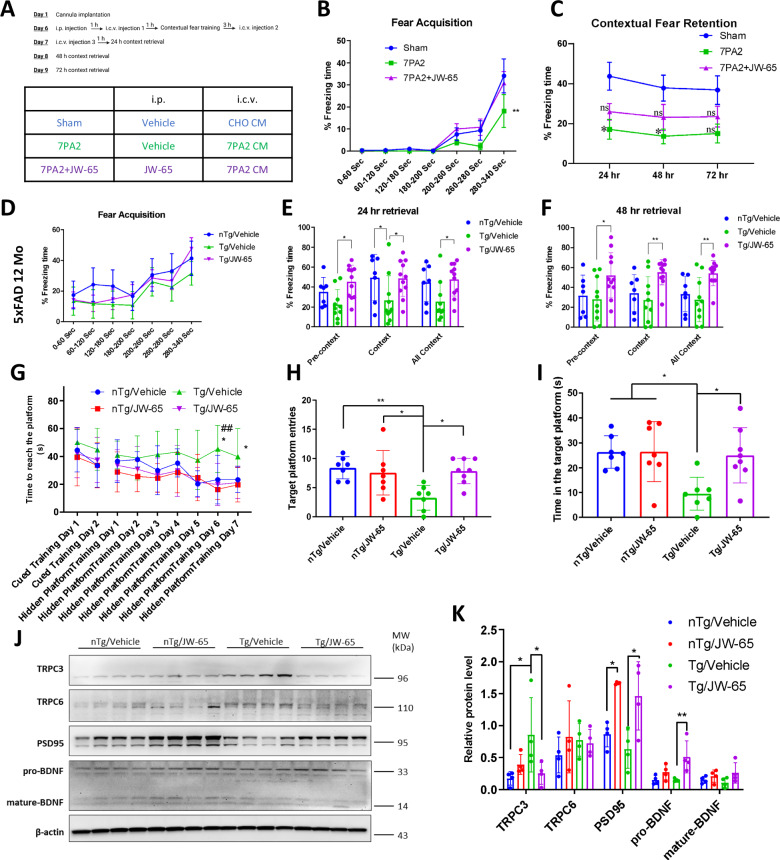
JW-65 treatment protects mice against Aβ toxicity in acute and chronic models. **A-F** JW-65 protect mice against 7PA2-impaired fear contextual memory. **A** The dosing schedule was based on our established model of the acute Aβ-induced synaptotoxicity [42]. The mice received one dose of JW-65 (20 mg/kg) or vehicle post-surgery through i.p. administration and three intracerebroventricular (i.c.v.) injections of 2 µL of 7PA2 CM or CHO CM. N = 7 mice for Sham, 4 for 7PA2, and 7 for 7PA2 + JW-65, respectively. **B** Fear acquisition during the training session (two-way ANOVA. **, p < 0.01 in comparison with Sham). **C** Summary of the 24-72 hr retrieval test (two-way ANOVA. ns, non-significant in comparison with Sham. (*, p < 0.05 in comparison with Sham). **D-F** Fear acquisition and 24-48 hr retrieval tests of 12-month-old 5xFAD mice (N = 7 mice for nTg/Vehicle, 10 for Tg/Vehicle, and 11 for Tg/JW-65, respectively, two-way ANOVA. *, p < 0.05. **, p < 0.01). **G** JW-65 treatment rescues memory in symptomatic 5xFAD mice. Water maze test was performed on mice at 8 months of age, with the JW-65 treatment group starting from 5 months of age. The time the mice spent reaching the platform during the cued training and hidden platform training sessions (N = 7 mice for nTg/Vehicle, 7 for nTg/JW-65, 7 for Tg/Vehicle, and 8 for Tg/JW-65, respectively. * note Tg/Vehicle vs nTg/Vehicle. ## notes Tg/Vehicle vs Tg/JW-65. *, p < 0.05, ##, p < 0.01). **H,I** Target platform zone entries and the time spent in the zone during the probe test (one-way ANOVA. *, p < 0.05. **, p < 0.01). **J,K** Expressional levels of TRPC3, TRPC6, PSD95, and BDNF proteins in hippocampal tissue (n = 4, two-way ANOVA. *, p < 0.05. **, p < 0.01).

We next used young adult WT B6 mice to test the optimal dose of JW65 for in vivo studies. As we reported [32], daily injection of JW65 compound intraperitoneally (i.p.) at 200 mg/kg dosage for five consecutive days resulted in no weight loss in B6 mice. Single i.p. injection of JW65 between 20 to 100 mg/kg all led to an increase in cortical and hippocampal PSD95 protein level on Western blots, indicating that JW-65 enhanced the postsynaptic scaffolding protein expression in excitatory neurons (Fig. S3A-B). Further analysis using both Western blots and RT-qPCR revealed that a dosage of 20 mg/kg led to a maximum increase in hippocampal PSD95/Dlg4 levels compared to the 10 mg/kg dosage (Fig. S3C-D). This dosage of 20 mg/kg was thus used in the subsequent acute and chronic in vivo experiments. We then tested the effect of JW-65 for hippocampus-dependent contextual memory in an acute AβOs model using 7PA2 CM as we described previously [40]. Following a single i.p. injection of JW-65 at 20 mg/kg, young adult WT B6 mice were injected (i.c.v.) with 2 μL of CHO control medium or 7PA2 CM through an implanted cannula (Fig. 3A). Contextual fear conditioning was then employed. Both context training and retrieval tests indicated that acute AβOs exposure impaired contextual learning and memory, as assessed by fear acquisition and retention processes. JW-65 administration prevented this memory impairment on young WT B6 mice (Fig. 3B, C). Together with the shRNA-downregulated TRPC3 data (Fig. 1L–N), these findings indicate that inhibition of TRPC3 improves cognitive and memory functions. This concept is further supported by bioinformatic gene analysis in the hippocampus relative to contextual fear memory across 22 strains of genetically diverse inbred BXD mice [63] revealed *Trpc3* being the sole member within the TRPC family that displayed an inverse correlation (Fig. S3E; *p* = 0.00862), supporting our finding that TRPC3 negatively regulates contextual memory.

In a control experiment, 7PA2 CM depleted by anti-Aβ antibody did not induce deficits in contextual memory (Fig. S3F), validating an AβOs-mediated synaptic mechanism as we previously described [40]. Intriguingly, JW-65 administration not only prevented the AβOs-impaired fear contextual memory on young adult B6 mice (Fig. 3B, C) but also rescued age-impaired fear contextual memory on older mice (Fig. S3G). These results indicate that inhibition of TRPC3 is not only effective for AD conditions but also beneficial for combating normal aging.

To evaluate the in vivo effect of JW-65, we investigated the therapeutic potential of chronic JW-65 treatment in the 5xFAD model. We administered symptomatic Tg mice (12-month-old) with 20 mg/kg i.p. twice weekly JW-65 over one month. Following this therapeutic intervention, fear conditioning was conducted to assess the efficacy of the treatment. The results demonstrated that Tg mice exhibited the expected phenotype of memory decline compared to their nTg littermates. However, this memory impairment was ameliorated following JW-65 treatment (Fig. 3D–F).

To further evaluate the therapeutic value of JW-65, we chronically administered the compound to a new cohort of symptomatic 5xFAD Tg and nTg littermate mice, using the same treatment regimen as previously described. We specifically selected symptomatic five-month-old 5xFAD Tg mice to initiate treatment, anticipating that the disparities between the phenotype and control mice would exhibit statistical significance. This age was chosen to ensure that the mice were not too advanced in age after three months of treatment to still be capable of swimming during the Morris water maze - a widely utilized behavioral test for evaluating spatial and related forms of learning and memory in rodents. Our results showed that, during the two training sessions, the Tg control group exhibited an inability to reach the platform or spent the longest average time in the water before reaching the destination (Fig. 3G), which indicate deficits in spatial learning and recognition. In contrast, JW-65 treatment significantly mitigated memory loss, as shown by the comparable times to reach the platform observed between the nTg mice and those dosed with JW-65 (Fig. 3G). In the probe test, the platform was removed, and all mice. were given 60 s to swim freely in the pool. All groups exhibited similar swimming distances, suggesting comparable locomotor activity. However, the Tg group displayed fewer entries and spent less time in the target zone. Tg mice treated with JW-65 demonstrated enhanced awareness of the correct platform location within the pool, with both time and entries comparable to the two nTg groups (Fig. 3H–I), suggesting that memory is improved upon TRPC3 inhibition.

Following the completion of the water maze, the mice were euthanized, and their hippocampal protein expression was examined. Notably, Tg control mice exhibited a markedly elevated expression of TRPC3 compared to their nTg littermates. Conversely, both PSD95 and BDNF showed a significant reduction in the Tg control group (Fig. 3J–K). Of note, all these changes were corrected by the JW-65 treatment. On the contrary, the TRPC6 level showed no significant alterations across all experimental groups (Fig. 3J, K). Consequently, we infer that JW-65 effectively inhibited and decreased the hippocampal TRPC3 level and concurrently enhanced the expression of neuroprotective proteins in the 5xFAD model.

### JW-65 restores impaired LTP and rescues synaptic gene expression in 5xFAD mice

**Fig. 4:**
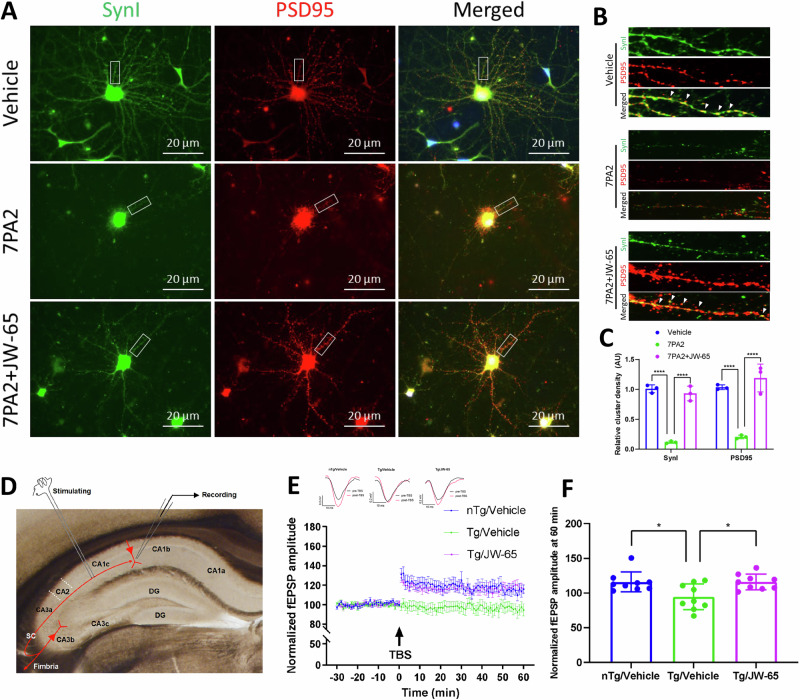
JW-65 improves synaptic performance in vitro and ex vivo. **A-C** Effects on active synaptic clusters. **A** Representative confocal images of immunosignals of synapsing I (SynI) and PSD95. **B**, Enlarged view of the SynI-PSD95 immunosignals on dendrites from the insets (white boxed area from panel A). The merged immunosignals represent colocalized SynI-PSD95 cluster and active synaptic zones (indicated by the white arrowheads) **C**, Quantification was performed similarly as for Fig. 1D, E: clusters of SynI-PSD95 (i.e., indicated by the white arrowheads in Panel B) on dendrites were counted, and the densities of synaptic clusters were calculated as the average number of clusters per primary dendrite. Data are presented as relative cluster density using the vehicle control as reference (value 1.0); two-way ANOVA. ****, p < 0.0001). **D-F** Ex vivo LTP recording on 5xFAD brain slices (13 months of age). **D** Pictorial illustration of the ex vivo LTP recording configuration. **E** Time course for theta-burst induced (black arrow) LTP depicting normalized fEPSP amplitude. n = 9. Top panel shows representative field synaptic responses collected during baseline (black line) and after theta burst stimulation (red line). Scale: 0.2 mV/10 ms. **F** Quantification of normalized fEPSP at 60 min post-TBS. (n = 9, one-way ANOVA. *, p < 0.05).

The data presented above strongly indicated that upregulated TRPC3 leads to memory dysfunction, which is reversed by JW-65. Thus, we next focused on whether protection by JW-65 is mediated through synaptic function. Indeed, the selective TRPC3 antagonist JW-65 was also found to restore the active synaptic zones/clusters impaired by AβOs as detected by the colocalized distribution of pre-synaptic synapsin I (SynI) and post-synaptic PSD95 as we previously described [39, 41]. Quantification based on the confocal images of dendritic spine structures with SynI-PSD95 clusters showed a significant protective effect from JW-65 (Fig. 4A–C). We then assessed the potential effect of JW-65 on rescuing the impacted LTP in symptomatic 5xFAD Tg transgenic mice. LTP is a paradigm directly associated with synaptic plasticity and learning/memory. Ex vivo LTP recordings were conducted on brain slices from middle-aged 5xFAD Tg mice. Field excitatory postsynaptic potential (fEPSP) was evoked in the proximal apical dendrites in hippocampal CA1b during the stimulation of Schafer-commissural projections in CA1a, and LTP was induced by theta burst stimulation (TBS) (Fig. 4D). Our observations revealed a significant reduction in TBS-induced LTP in old transgenic mice, suggesting an impacted LTP in the Tg mice. Conversely, the JW-65 treatment completely normalized the impaired LTP, with the fEPSP level being restored to a comparable level as observed in nTg control mice (Fig. 4E, F), suggesting a complete recovery of the impaired LTP. Interestingly, chronic JW-65 treatment did not seem to exert significant effects on lowering amyloid load, including both soluble Aβ and plaques, and reducing gliosis in mid-aged mice (Fig. S4A-C), despite a notable impact on restoring neuronal loss and dendritic spines, as indicated by Golgi staining (Fig. S4D-E).

**Fig. 5:**
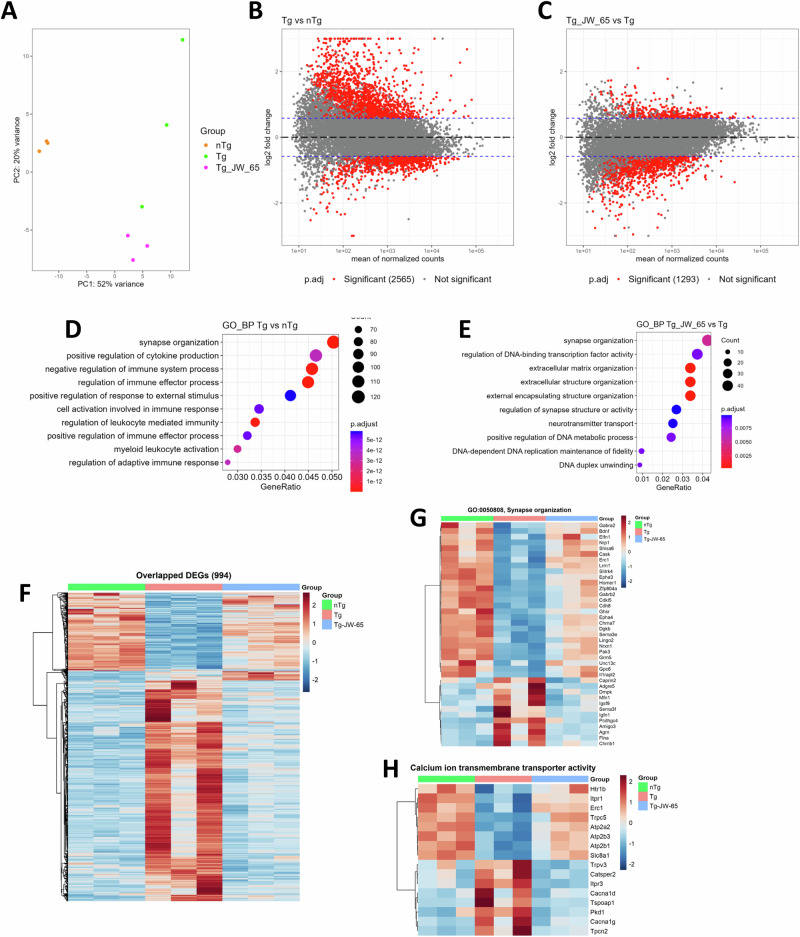
RNA-seq data analysis. **A** PCA plot of the RNA-seq data from hippocampal tissue isolated from the brains of 5xFAD mice (N = 3 mice each group; 12 months of age). **B** MA plot of the comparison between Tg mice and nTg mice. Red dots on the graph represent statistically significant data points. *p.adj* < 0.05 and LFC > 0.58. **C** MA plot of the comparison between Tg mice received JW-65 treatment and Tg control mice. Red dots on the graph represent statistically significant data points. *p.adj* < 0.05 and LFC > 0.58. **D,E** GO enrichment column graph showing the number of genes that are significantly different in Tg mice compared with nTg mice and untreated Tg compared to JW-65-treated Tg regarding the biological process (BP), related GO terms. **F-H** Heatmaps showing the critical genes altered (*p.adj* < 0.05 and LFC > 0.58) by JW-65 treatment in Tg mice.

To further uncover the molecular mechanisms underlying the synaptic protective effect of JW-65 and TRPC3 inhibition, we conducted RNA-seq experiments to capture global gene expression patterns using hippocampal total RNA from middle-aged mice. Principal component analysis (PCA) demonstrated a clear separation among the three groups, with a major variance (PC1 = 52% variance) between the nTg group and the two Tg groups (Fig. 5A). Further analysis revealed that 2565 genes exhibited significant differences between the Tg and nTg control littermates (*p.adj* < 0.05 and Log2 Fold Change (LFC > 0.58) (Fig. 5B). Tg mice in the JW-65 treatment group displayed 1293 differentially expressed genes compared to Tg control mice (Fig. 5C). Gene ontology (GO) analysis was employed to gain insights into the functional implications of the identified differentially expressed genes (DEGs). The GO analysis of BP highlighted major and significant enrichment of genes associated with synapse organization in Tg mice and immune response, indicating a prominent feature of neuroinflammation in the 5xFAD model (Fig. 5D, E). The 994 overlapped DEGs were identified between the two compared groups, and most of those DEGs were reversed by JW-65 compared to Tg mice, including synapse organization genes like *Bdnf* as well as genes associated with Ca^2+^ ion membrane transporter activities (Fig. 5F–H and Fig. S5A). These findings are consistent with the transcriptomic data from human AD brains showing synaptic genes are largely downregulated [64–67]. Interestingly, the several major categories of microglia-associated genes including the disease-associated microglial DAM or microglial neurodegenerative phenotypes (MGnD) genes (*ApoE*, *Cst7*, *Clec7a*, Ccl2, *Ccl6*, *Trem2*, *Tgf1b, Gpnmb, Itgax, Csf1*, and *Spp1*), and the homeostatic microglial genes (*Aif1* and *Tmem119*) [68, 69] were all significantly increased in Tg mice. However, none was significantly affected by JW-65 (Fig. S5B-D). It is worth noting that the expression of the *Trpc3* gene was slightly increased (LFC = 0.473) and significantly decreased with JW-65 treatment (LFC = −0.776, *p.adj* = 9.96E-3) (Fig. S5A). On the contrary, *Trpc4* and *Trpc5* genes were significantly decreased in Tg but restored by JW-65 (Fig. S5A). Of note, reduced expression of TRPC4/5 have been shown to be associated with impaired synaptic plasticity and spatial working memory formation [70], though it is unclear how JW-65 can restore their gene expression, presumably via corrected Ca^2+^ signaling. Taken together, these findings strongly support synaptic protection being the primary mechanism mediating the in vivo efficacy of JW-65 in the 5xFAD model.

### JW-65’s synaptic protective mechanism is mediated by the Ca^2+^-dependent calmodulin kinase (CaMK) pathways

We next investigated the downstream cell signaling pathways underlying the neuroprotective effect of JW-65 by co-incubating cell cultures with a panel of kinase inhibitors commonly associated with the neuroprotective pathways. While inhibitors such as RP-cAMP (cyclic adenosine monophosphate inhibitor), LY294002 (phosphoinositide 3-kinase inhibitor), and U0126 (mitogen-activated protein kinase inhibitor) showed no significant effects, KN93, a Ca^2+^/calmodulin-dependent protein kinases inhibitor, abolished the neuroprotective effect from JW-65 (Fig. S6A-B); KN93 treatment alone also induced significant neurotoxicity like 7PA2 while its inactive counterpart KN92 had no effect (Fig. S6C). Furthermore, JW-65- treatment restored CaMKII and CaMKIV activities in specific phosphorylated forms as observed using Western blots: Thr286-CaMKII and Thr196-CaMKIV (Fig. S6D-E). These results suggest that the Ca^2+^/calmodulin-dependent mechanism mediates the neuroprotective action of TRPC3 antagonist against AβOs-induced synaptic toxicity. Additional Western blot and RT-qPCR results also displayed a corrective effect from JW-65 on restoring levels of BDNF and PSD95, and reduced expression of the cleaved caspase-3, a marker for apoptosis (Fig. S6F-H).

### TRPC3 contributes to Ca^2+^ overload during AβOs-induced neurotoxicity

**Fig. 6:**
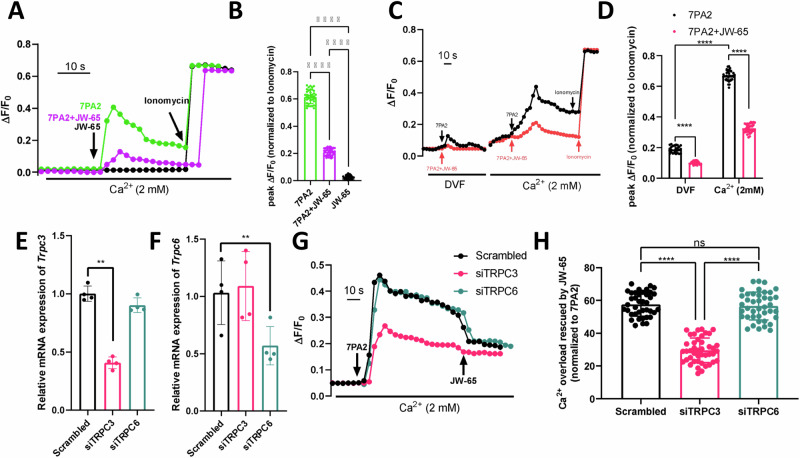
TRPC3 contributes to AβOs-induced neuronal Ca^2+^ overload. **A** Representative fluorescent recordings on neurons perfused with 7PA2, JW-65, or their combination, with the presence of extracellular Ca^2+^. **B** Quantification of the peak cytosolic Ca^2+^level induced by 7PA2, normalized to ionomycin-induced overload (n = 38 for 7PA2, 35 for 7PA2 + JW-65, and 32 for JW-65, respectively, one-way ANOVA. ****, p < 0.0001). **C** Representative fluorescent recordings on neurons perfused with 7PA2 or in combination with JW-65 within a divalent-free (DVF) environment. The cells were then washed and exposed to an extracellular solution containing Ca^2+^ and perfused again with 7PA2 or in combination with JW-65. **D** Quantification of the peak cytosolic Ca^2+^ level induced by 7PA2, normalized to ionomycin-induced overload (n = 27 neurons for DVF and 32 neurons for Ca^2+^-added conditions, respectively, two-way ANOVA. ****, p < 0.0001). **E,F** RT-qPCR validation of the knock-down of TRPC3 and TRPC6 (n = 3, one-way ANOVA. **, p < 0.01). **G** Representative fluorescent recordings on scrambled control, siTRPC3, and siTRPC6 neurons, with the presence of extracellular Ca^2+^. 7PA2 was perfused first to induce Ca^2+^ overload. JW-65 was then perfused to study its effects. **H** Quantification of the Ca^2+^ overload rescued by JW-65, normalized to 7PA2-induced overload (n = 39 for Scrambled, 43 for siTRPC3, and 41 for siTRPC6, respectively, one-way ANOVA. ns, non-significant. ****, p < 0.0001).

In healthy neurons, cytosolic Ca^2+^ concentration is typically maintained at a lower level compared to the extracellular space or certain intracellular compartments. However, in AD, the presence of Aβ disrupts Ca^2+^ signaling by inducing an increase in intracellular Ca^2+^ levels, known as Ca^2+^ overload [70]. We conducted calcium imaging on primary cultured neurons at DIV13 to visualize the dynamics of Ca^2+^. In the presence of extracellular Ca^2+^, 7PA2 induced an increase in cytosolic Ca^2+^ level (Fig. 6A). Perfusion of 10 µM ionomycin at the end of the recording was used to provide a standard reference for Ca^2+^ overload. Notably, co-perfusion of JW-65 (500 nM) with 7PA2 significantly alleviated Ca^2+^ overload (Fig. 6A, B). Moreover, when cells were recorded in a divalent-free (DVF) extracellular environment, 7PA2 could still increase the cytosolic Ca^2+^ level, albeit the increase was significantly lower than the increase observed when Ca^2+^ was supplemented (Fig. 6C, D). This suggests that 7PA2 can induce both Ca^2+^ store-release and influx, ultimately resulting in Ca^2+^ overload. Of note, JW-65 treatment significantly prevented both Ca^2+^ store-release and influx induced by 7PA2, as indicated by more than 50% reduced fluorescence readout in both solutions (Fig. 6C, D).

To confirm whether the effect of JW-65 is mediated through TRPC3, we transfected rat primary neurons with siRNAs specifically targeting TRPC3 (siTRPC3) or TRPC6 (siTRPC6), respectively, 48 h prior to recording. Successful knockdown was validated by RT-qPCR (Fig. 6E, F). In all cases, 7PA2 elevated intracellular Ca^2+^ concentration. However, the elevation of Ca^2+^ was significantly lower when TRPC3 was knocked down (Fig. 6G, H). Furthermore, JW-65 only exerted a minor effect when the expression of TRPC3, but not TRPC6, was knocked down (Fig. 6G, H), indicating TRPC3 as its true target. While it is established that Aβ induces Ca^2+^ overload by interacting with various ion channels, our results indicate that TRPC3 is also significantly involved in this process.

## Discussion

We report here that chronic application of a newly developed pharmacological antagonist compound JW-65 completely rescues synaptic dysfunction and learning memory deficits in symptomatic 5XFAD Tg mice. Given the well-recognized concept that AD is a disease of synaptic failure [71, 72], the findings disclosed in this work not only identify a promising pharmacological agent with translational value but also pinpoint several potential mechanistic insights of TRPC3 uniquely contributing to the pathogenesis and progression of AD.

### An inverse correlation between TRPC3 expression and aging/AD - aberrantly upregulated TRPC3 in glutamatergic excitatory neurons

We report here that TRPC3 expression is elevated in the hippocampus of both aged and 5xFAD Tg mice brains. By immunohistochemistry, TRPC3 expression was found to be aberrantly upregulated in 4 out of the 4 postmortem AD frontal forebrain specimens with high levels of amyloid pathology, compared to the control cases (with low amyloid burden), most notably in the large excitatory pyramidal neurons (Fig. 1A–C). This finding suggests an association between increased levels of TRPC3 with high amyloid burden. Indeed, a significant association was initially identified between TRPC3 expression in the prefrontal cortex of AD cases positive for neuritic amyloid plaques based on the ROS/MAP data, but no association found with neurofibrillary tangles, cerebral amyloid angiography, or Lewy bodies [73].

In primary cultured hippocampal neurons derived from embryonic E17 rats, we recently reported [40] that TRPC3 is upregulated by 7PA2 treatment, primarily in the excitatory neurons expressing high level of CaMKIIa at the transcriptional level via Ca^2+^-induced calcineurin (CaN)-coupled Nuclear factor of activated T-cells (NFAT) activation. TRPC3 expressional control is reportedly to be subjected to feedback regulation at both transcriptional and protein levels. This AβOs-induced Ca^2+^ overload, partially as a result of an increased TRPC3 channel activity, contributes to a feedforward loop that upregulates its own expression in AD neurons. We believe that JW-65’s dual effects in preventing TRPC3 upregulation, as well as in restoring the downstream cellular signaling events (e.g., CaMK-dependent) are all attributed to its ability of restoring the Ca^2+^ signaling.

TRPC3 is widely expressed in almost all cell types in the CNS. Although we observed upregulated TRPC3 expression in primary cultured astrocytes and microglia [40], the notion that chronic JW-65 treatment lacks anti-inflammatory effect in 5xFAD Tg mice argues against TRPC3’s significant roles in the glial cells during AD progression in this mouse model (Fig. S5). Potentially differentiated expression relating TRPC3 functions in different cell types in CNS, especially in white matter, during AD pathogenesis warrants further investigation.

### How does the upregulated TRPC3 expression in excitatory neurons contribute to synaptic failure and AD pathogenesis: hyperexcitability vs hypoexcitability?

Through testing the selective TRPC3 antagonist compound JW-65 in the chronic 5xFAD mouse model, we learned that aberrantly upregulated TRPC3 negatively correlates with impaired synaptic morphology and functions. Moreover, the results corroborate well between our ex vivo AβOs-challenged neuronal culture model and hippocampal slice recording, demonstrating that TRPC3 overactivation can contribute to AD synaptic failure in conjunction with amyloid pathology. Interestingly this forms a vicious cycle, where AβOs induce TRPC3 upregulation, due to Ca^2+^ overload, which contributes to sustained Ca^2+^ dyshomeostasis. Here, we demonstrate that JW-65 treatment can rescue impaired LTP in the hippocampus of 5xFAD Tg mice, largely restoring expression of the impaired synaptic genes as well as the gene clusters involved in the Ca^2+^ signaling pathways (Fig. 6).

A question is raised of what the upregulated TRPC3 expression does to impair synaptic functions. In our recent report [40], we demonstrated that overexpressing TRPC3 in the mouse hippocampal CA1 resulted in neuronal hyperexcitability when determined several weeks after viral expression in young adult mouse brains. This is reminiscent of the hyperexcitability often detected in the early stage of clinical AD [74, 75]. Interestingly, overactivated TRPC3 channels in Purkinje neurons not only underlies the moonwalk ataxia [27, 28], it is also associated with seizure [24, 25]. Our recent report [76] demonstrates a potent anti-seizure effect of the same TRPC3 antagonist compound JW-65 in mice, strongly arguing for a significant role of overactivated TRPC3 in hyperexcitability.

Although seizures were not detected in 5xFAD Tg mice at any age, hyperactivity was reported to be the most notable behavioral phenotype of the mouse model [77]. Surprisingly, hyperexcitability was only reported in 5xFADTg in old age in one study [78]. On the contrary, an earlier report suggested that insufficient learning-related intrinsic excitability (hypoexcitability) underlies synaptic failure in middle-aged 5xFAD Tg mice [79], this was further attributed to an increase in Ca^2+^-sensitive spike afterhyperpolarization (AHP) which could be corrected by TRPC3 blockage [29]. Taken together, we speculate that overactivated TRPC3 first causes hyperexcitability in mice, which may ultimately lead to hypoexcitability and synaptic failure (e.g., LTP) in old age. Future work should be carefully designed to test this hypothesis and provide mechanistic distinctions of TRPC3 overactivation in altering synaptic performance in the hippocampus.

### How does JW-65 work in halting AD?

Our presented data indicates that the upregulation of TRPC3 protein expression contributes to synaptic failure and impaired learning/memory, and thus may play a crucial role in AD pathogenesis and progression. The significant synaptic protective effect of JW-65 treatment not only corrected/prevented cognitive impairment in the 5xFAD model, but it also normalized the aberrantly upregulated TRPC3 expression. Although Trpc genes are not among the identified DEGs, the symptomatic 5xFAD Tg hippocampal tissue display increased expression of Trpc3 gene which was significantly restored by the JW-65 treatment (Fig. S6A). The bulk hippocampal RNAseq data analysis also identifies multiple genes related to Ca^2+^ homeostasis/signaling that are impaired in the 5xFAD Tg compared to littermate nTg which are largely corrected by the JW65 treatment (Fig. 6). We therefore speculate that JW-65’s effect to correct upregulated neuronal expression of TRPC3 is primarily owing to its ability to restore Ca^2+^ homeostasis after AβOs challenge and thus correcting the overactivated Ca^2+^-dependent CaN-NFAT signaling, resulting in normalized *Trpc3* gene expression at transcriptional level. Interestingly, JW-65’s beneficial effect seems to be not limited to AD model mice, its treatment also improves hippocampus-dependent fear contextual memory in normally aged mice (Fig. S3G). It is unclear why and how JW-65 treatment could restore the aberrantly upregulated TRPC3 expression, acutely in cultured neurons and chronically in mice, in both normally aged and symptomatic 5xFAD Tg mice. It is plausible to attribute the rescuing mechanism to the restored Ca^2+^ homeostasis which governs the CaN-NFAT signaling. Although Aβ amyloid pathology does not generally develop during normal aging (by definition, Aβ pathology is what characterizes AD pathology), additional neurotoxic mechanisms (e.g., oxidative stress and heavy metals) can also upregulate TRPC3 expression via similar mechanism of dysregulated Ca^2+^ homeostasis. On a separate note, although we could not exclude JW-65’s potential off-target effect in synaptic protection based on the data we collected, the results presented using both the TRPC3 KO neurons (Fig. 2F, G) and *Trpc3*-selective siRNA (Fig. 6G) provide strong evidence supporting the TRPC3 target-based mechanism(s).

### Ca^2+-^dependent synaptic signaling pathways - TRPC3-BDNF axis in AD

In the brain, calcium is fundamental in the control of synaptic activity and memory formation, a process that is mediated via transducing the Ca^2+-^dependent signals through CaMKs to the activation of protein effectors such as transcription factor CREB and its most important gene target encoding for BDNF [80–83]. Given the prominent roles of BDNF in regulating synaptic plasticity and the cognitive functions of the brain, we reason that JW-65 rescues hippocampal LTP at least partially via restoring BDNF functions. Indeed, the JW-65 treatment restored *Bdnf* gene transcription in cultured neurons and 5xFAD hippocampal tissues. Paradoxically, neither the cAMP analog, nor PKA inhibitor could interfere with JW-65’s protective effect, indicating a pathway independent of the canonical cAMP/PKA-CREB-BDNF signaling. Instead, JW-65’s protective effect is dependent on the Ca^2+^/calmodulin-mediated mechanisms involving CaMKII/CaMKIV activation.

Interestingly, all known excitatory neuron subtypes express high levels of BDNF. Although impaired BDNF signaling has been linked with increased amyloid and tau pathology, neuroinflammation, and neuronal loss, the exact mechanisms underlying the effect of impaired BDNF signaling on AD are still largely unknown [82]. BDNF signaling through its major receptor TrkB requires Ca^2+^ influx and the activation of Ca^2+^/CaMKII [82]. Therefore, our data supports a theory that defective BDNF signaling in AD may be a result of dysregulated Ca^2+^ and the Ca^2+^/calmodulin-dependent signal transduction in which TRPC3 overactivation may play a part. On the other hand, BDNF-induced TrkB activation mediates phospholipase C (PLC)-dependent TRPC3 channel activity and Ca^2+^ entry in pontine neurons during development [84]. In addition, TRPC3 channel activity is essential for nerve-growth-cone guidance by BDNF in cerebellar granule cells [85]. Of particular interest, TRPC3 was found to be required for the BDNF-TrkB-induced slow depolarization in CA1 pyramidal neurons to promote dendritic spine density [86]. Moreover, BDNF from mossy fibers evokes a TRPC3 current and Ca^2+^ elevations in CA3 pyramidal neurons [87]. All these prior reports identify TRPC3 as a, if not the, mediator of BDNF-induced membrane current channel, acting via TrkB under physiological conditions. On the contrary, our work discloses a novel association between aberrantly upregulated TRPC3 and impaired BDNF signaling during AD progression, both may be attributed to Ca^2+^ overload, which potentially involves overactivated TRPC3 channel. Of note, the current knowledge indicates the seemingly opposing roles of TRPC3 in basal synaptic transmission (e.g., impaired CA1 LTP in young adult TRPC3KO mice [88], and the upregulated TRPC3 in AD pathogenesis.

### Neuronal calcium signaling as a therapeutic target for AD - targeting TRPC3 or TRPC6 or both?

There is extensive previous literature suggesting that dysregulated Ca^2+^ signaling plays a key role in AD pathogenesis and that Ca^2+^ signaling modulators may have potential neuroprotective effects in AD [89, 90]. The targets that were previously considered in this context include RyR2 [91] and our recent genetic studies demonstrated that increased basal activity of RyR2 inhibits autophagy in AD neurons [92]. Another potential target is CaN. Retrospective analysis of epidemiological data revealed a significantly reduced incidence of AD in organ transplant patients chronically treated with CaN inhibitor FK-506 [93]. A recent report based on a large, diversified patient population with controls for age, sex, race, and multiple AD risk factors disclosed that the use of the FDA-approved CaN inhibitor, tacrolimus is also associated with reduced prevalence of dementia-related symptoms [94]. Our recent results suggested that positive allosteric modulators of SERCA Ca^2+^ pump (NDC compounds) may also stabilize neuronal Ca^2+^ signaling and exert neuroprotective effects in AD models [95, 96]. In the present study, we report that similar synaptoprotective and neuroprotective effects can be achieved by selective TRPC3 inhibitor JW-65.

**Fig. 7:**
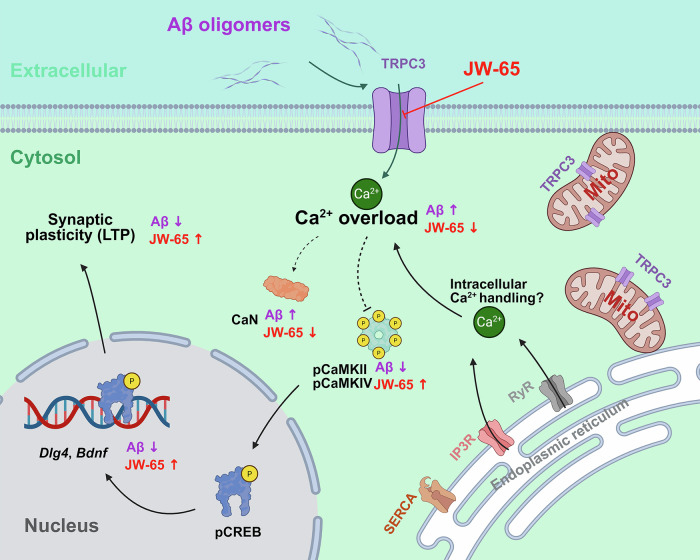
Graphical abstract. Schematic summary of our findings of the novel contribution of TRPC3 in Ca^2+^ signaling in synaptic plasticity and its aberrant roles in mediating Ca^2+^ overload induced by Aβ oligomers. TRPC3 plays significant roles contributing to Ca^2+^ overload through both entry from the plasma membrane and the store-released mechanisms. Of note, the subsequent cell signaling events downstream of the Ca^2+^ overload, including impaired CaMKII/IV activities, along with the downregulated synaptic effector genes (e.g., *Bdnf* and *Dlg4*), as well as the overactivated calcineurin (CaN) can be corrected and restored by TRPC3-selective antagonist compound JW-65. An outstanding question remains as whether TRPC3-mediated Ca^2+^ entry is through SOCE; the colocalization of TRPC3 in the inner membrane of the mitochondria [100] implicates a potential involvement of TRPC3 in intracellular Ca^2+^ handling (e.g., between ER and mitochondria). How TRPC3 is involved in modulating SERCA (ER Ca^2+^-ATPase) expression at the ER membrane and their potential interplay with the inositol 1,4,5-triphosphate receptors (InsP_3_Rs) or ryanodine receptors (RyRs) in Ca^2+^ release from ER in stressed neurons also warrants further investigation. Figure was created using BioRender.

As illustrated in Graphical abstract (Fig. 7) with AβOs agents used to challenge neurons in an AD model, inhibitors of TRPC3 and other Ca^2+^ signaling modulators may act by preventing Ca^2+^ overload via suppressing the overactivation of CaN as well as preserving the calmodulin-dependent kinases CaMKs, resulting in synaptoprotection. Because of these convergent mechanisms of action, the aforementioned compounds may also exert synergistic effects when used in combination.

In earlier reports, TRPC3/6 were often reported together due to overlapping roles in physiological functions, such as promoting cerebellar granule neuron survival [17]. Although both are highly expressed in CNS in the hippocampus, they appear to be expressed in different hippocampal subregions [8–10], implying that they may play distinct roles in various neuronal subtypes. More recently, divergent roles of TRPC3 and TRPC6 have been increasingly reported especially in neuronal hyperexcitability and epilepsy [24, 25]. Of note, TRPC6 has been most studied in AD to be neuroprotective, and pharmacological TRPC6 agonists such as hyperforin and piperazine derivatives have also been reported to be promising agents for treating AD [1, 97, 98]. A recent proteomic study indicates that TRPC3/6 frequently form in homomers while TRPC1/4/5 are in various heteromers [99]. Given that TRPC3 and 6 heterodimers have never been identified thus far under physiological conditions, we speculate that they may be expressed in different neuronal subpopulations. Differential distributions of these two members in different intracellular compartments, such as TRPC3 in mitochondria [100] and TRPC6 in endoplasmic reticulum/ER [20] suggest their potentially distinct roles in intracellular Ca^2+^ handling, which warrants further investigation. A fuller understanding of the potentially distinct roles that each TRPC member plays during disease onset and progression is imperative and instrumental for rationalizing drug targeting strategies and treatment options. Ultimately, the choice of Ca^2+^ signaling modulators for future therapeutic development will be determined by their relative efficacy and safety profiles.

## Supplementary information

Supplementary File

## Data Availability

Raw and processed RNA-seq data used in this study are available at the Gene Expression Omnibus under the accessions GSE253510.
