# Supplementary material for: Pyrazole-derived TRPC3 antagonist ameliorates synaptic dysfunctions and memory deficits in Alzheimer’s disease models
Source: Mol Psychiatry. 2026 Apr 11;31(9):5031–47. doi: 10.1038/s41380-026-03592-6 (PMC13441893; doi:10.1038/s41380-026-03592-6)
Supplement: Supplementary file 1 [file 41380_2026_3592_MOESM1_ESM.docx]

Supplementary Materials for

**Pyrazole-derived TRPC3 antagonist ameliorates synaptic dysfunctions and memory deficits in Alzheimer’s disease models**

Jiaxing Wang *et al.*

*Corresponding author. Email: [fliao@uthsc.edu](mailto:fliao@uthsc.edu)

**This PDF file includes:**

Figs. S1 to S6

Tables S1 to S3

**Figure S1. Increased expression of TRPC3 in normally aged WT B6 and 5xFADTg mice. A**, TRPC3 protein levels in WT mice at three ages as detected by Western blots and quantified based on three independent experiments (one-way ANOVA. **, p<0.01. ***, p<0.001). **B**, Representative images of TRPC3 immunosignals from immunohistochemistry using Alomone’s rabbit antibody (ACC-016, 1:1000): young and old brains correspond to 2 and 20 months of age of male WT B6 mice. DG: dentate gyrus. **C**, Trpc3 mRNA profile by in situ hybridization of the same WT mice at 2 and 20 months of age. **D**, Representative image of amyloid-β immunohistochemistry using Sigma’s antibody (BAM-10, 1:1000) of 5xFAD littermate mice (8 month-old). **E**, Representative image of in situ hybridization of the same pair of 5XFAD mice presented in panel D.

**Figure S2. Effects of JW-65 compound treatment on neuronal morphology. A**, Representative microscopic images showing that 7PA2 dose-dependently induces neuronal loss as evidenced by MAP2 staining (18 hr after 7PA2) on treated primary rat neurons at 14 DIV. **B,** Representative Western blots showing effect of JW-65 treatment in preventing 7PA2-induced TRPC3 upregulation in primary rat neurons at 14 DIV. **C,** Quantification based on Western blots from two independent experiments (two-way ANOVA). *P<0.05). **D**, Representative confocal images of the WT and APP-KI mouse hippocampal neurons transfected with TD-Tomato and treated with different concentrations of JW-65 on DIV15 and fixed at DIV16. **E**, Quantification of the percentage of the mushroom spines based on the images taken in the same experiments of panel **D** (from left to right respectively n=20, 20, 19, and 19 neurons, collectively from 3 batches of cultures, one-way ANOVA. ****, p<0.0001).

**Figure S3. A-D**, **Dose response of JW-65 treatment on the expression of synaptic protein PSD95 in WT mice.** **A-B,** PSD95 expression is enhanced in both the hippocampus and cortex of 4-month-old WT mice post one i.p. injection of 20/50/100 mg/kg JW-65. **C-D**, Western blot and RT-qPCR indicate IP administration of 20 mg/kg of JW-65 more significantly increased hippocampal PSD95 (*Dlg4*) level compared to 10 mg/kg. **E,** TRPC3 expression level is negatively correlated with fear contextual memory. *Trpc3* transcript expression from the hippocampus of 22 recombinant inbred strains (BXD type) revealed a negative relationship between its expression and contextual fear memory (Pearson correlation, r = −0.539, *p* = 0.0086). **F,** Fear contextual memory retrieval test with 7PA2 CM, vehicle (CHO CM) and Aβ-depleted 7PA2 CM (n= 7-9 per group of young adult B6 mice (two-way ANOVA. P<0.05 and **P<001). **G,** Effect of JW-65 treatment (4 i.p. injections, at 20 mg/kg, every other day) in rescuing impaired contextual memory in older B6 mice (15 months of age, n= 8-10 per group, two-way ANOVA. P<0.05 and **P<001).

**Figure S4. JW-65’s effects on AD pathological hallmarks in treated 5xFAD mice (n=3 mice each genotype). A,** Chronic JW65 treatment did not lead to weight loss. **B-C,** Representative images and quantification of IHC staining of anti-Aβ antibody, anti-Iba1 and anti-GFAP (n=3, two-way ANOVA. “ns”: non-significance. ***, p<0.001. ****, p<0.0001). **D,** Soluble Aβ42 concentrations determined by ELISA (n=6 samples/group; one-way ANOVA. “ns”: non-significance. **E-F**, Representative images and quantification of Golgi staining of the hippocampus in 5xFAD mice (N=3 mice at 12 months of age, one-way ANOVA. ****, p<0.0001).

**Figure S5. Bulk RNA-seq data from hippocampal tissue (N=3 5xFAD mice, 13 months of age). A**, Relative gene expression levels encoding for the TRPC family members and BDNF. **B**, Heatmap of microglia-associated genes. **C-D**, Relative gene expression levels for the Disease-Associated Microglia (DAM) and homeostasis genes as well as gene encoding for two complement C3.

**Figure S6. Cell signaling pathways underlying neuroprotective JW-65 in 14 DIV neurons.** **A**, Representative MAP-2 images show the neuroprotection of JW-65 was counteracted by KN93 (10 µM), a CaMKII inhibitor, but unaffected with the co-treatment of other kinase inhibitors, including RP-cAMP (10 µM), LY294002 (10 µM), and U0216 (10 µM). **B**, Quantification of dendritic length based on MAP-2 staining under the co-treatment with different kinase inhibitors (From left to right, n=22, 18, 25, 24, 25, 33, and 30, respectively. N=3 independent cultures, one-way ANOVA. “ns”: non-significance in comparison with Vehicle. ****, p<0.0001 in comparison with Vehicle). **C**, MAP-2 Immunostaining of primary neurons treated with KN92 (10 µM) or KN93(10 µM). **D**, Representative Western blots showing the phosphorylation levels of CaMKII and CaMKIV in hippocampal neurons and cortical neurons at various time points. **E**, Three independent blots are quantified based on the 2 hr time point of the panel D experiments (n=3, two-way ANOVA. *, p<0.05. **, p<0.01). **F**, Western blots showing the expressional levels of PSD95, BDNF, and cleaved caspase-3 in DIV14 neurons treated with or in combination with JW-65. **G**, The blots are quantified (n=2 for Control, 2 for 7PA2, and 4 for 7PA2+JW-65, two-way ANOVA. *, p<0.05. **, p<0.01, ***, p<0.001. ****, p<0.0001). **H**, mRNA levels of *Dlg4* and *Bdnf* in the same cohort of neuronal samples in panel **G** (n=3, two-way ANOVA. *, p<0.05. **, p<0.01).

**Table S1. List of human brain cases used in immunohistochemistry.**

|  | Sample ID | Age | Race | Gender | Braak | β-amyloid levels | PMI (hr) | Diagnosis |
| --- | --- | --- | --- | --- | --- | --- | --- | --- |
| NC | 5293 | 88 | white | M | I | Low | 2.67 | VaD+ARTAG |
| NC | 5077 | 80 | white | F | III | Low | 5 | VaD+ARTAG |
| NC | 5062 | 92 | white | M | II | Low | 2 | Normal+multiple infarcts |
| NC | 1304 | 81 | white | M | 0 | Low | 4.83 | Normal + ARTAG |
| NC | 1314 | 86 | White | F | I | Low | 4.83 | Normal |
| AD | 5272 | 78 | white | F | VI | High | 5.5 | ADNC+CVD |
| AD | 1068 | 92 | white | F | VI | High | 2 | ADNC+DLB+CVD |
| AD | 5079 | 85 | white | F | VI | High | 6 | ADNC +CVD |
| AD | 5371 | 93 | white | F | V | High | 4.8 | ADNC +CVD |

NC: non-AD cases

VaD: Vascular Dementia

ARTAG: Arteriosclerosis-Atherosclerosis

CVD: Cardiovascular Disease

DLB: Dementia with Lewy Bodies

ADNC: Alzheimer’s Disease with confirmed Neuropathologic Changes

**Table S2. List of primers used in this research.**

| **Primer** | **sequence** |
| --- | --- |
| *mTrpc3*, forward | TTAATTATGGTCTGGTTCTTGG |
| *mTrpc3*, reverse | TCCACAACTGCACGATGTACT |
| *mTrpc6*, forward | GCAGCTGTTCAGGATGAAAC |
| *mTrpc6*, reverse | TTCAGCCCATATCATGCCTA |
| *mDlg4*, forward | TCTGTGCGAGAGGTAGCAGA |
| *mDlg4*, reverse | CGGATGAAGATGGCGATAG |
| *mBdnf*, forward | AATTAAGCTTCCAATCGAAGCTCAACCG |
| *mBdnf*, reverse | AATTGAAATTCTCCACACAAAGCTCTCGGA |
| *mGapdh*, forward | GCAAATTCAACGGCACAG |
| *mGapdh*, reverse | CTCGCTCCTGGAAGATGG |
| *rTrpc3*, forward | CCACATGCAGTGAGACTTTGACTC |
| *rTrpc3*, reverse | AGGCCAACCTTGGGATCATTT |
| *rTrpc6*, forward | AGAAATTTGGAATTTTGGGAAGTC |
| *rTrpc6*, reverse | TCCTTATCAATCTGGGCCTGC |
| *rDlg4*, forward | GGCACACAAGTTCATTGAGG |
| *rDlg4*, reverse | GAGACATCGAGGATGCAGTG |
| *rBdnf*, forward | AGCGCGAATGTGTTAGTGGT |
| *rBdnf*, reverse | GCAATTGTTTGCCTCTTTTTCT |
| *rbActin*, forward | CCCGCGAGTACAACCTTCT |
| *rbActin*, reverse | CGTCATCCATGGCGAACT |

**Table S3. List of antibodies used in this research.**

| **Antibody** | **Source** | **Cat#** |
| --- | --- | --- |
| TRPC3 | Alomone | ACC-016 |
| TRPC3 | Abcam | ab51560 |
| TRPC6 | Alomone | ACC-017 |
| TRPC6 | Sigma-Aldrich | SAB4300572 |
| PSD95 | Thermo Fisher | 51-6900 |
| BDNF | Alomone | ANT-010 |
| pCaMKII | Cell signaling | 12716 |
| CaMKII | Cell signaling | 3362 |
| pCaMKIV | Santa Cruz | sc-28443-R |
| CaMKIV | Santa Cruz | sc-166156 |
| Cleaved caspase-3 | Cell signaling | 9611 |
| β-actin | Sigma-Aldrich | A2228 |
| Synapsin 1 | Sigma-Aldrich | A-6442 |
| IBA1 | WAKO | 019-19741 |
| GFAP | Sigma-Aldrich | G3893 |
| BAM-10 (β-Amyloid) | Sigma-Aldrich | A5213 |
| Goat anti-Mouse HRP | Thermo Fisher | 31430 |
| Goat anti-Rabbit HRP | Thermo Fisher | 31460 |
| Goat anti-Mouse Alexa Fluor Plus 488 | Thermo Fisher | A32723 |
| Goat anti-Rabbit Alexa Fluor Plus 594 | Thermo Fisher | A21207 |
